# Collectanea Medica

**Published:** 1813-09

**Authors:** 


### COLLECTANEA MEDICA,

CONSISTING OF

### ANECDOTES, FACTS, EXTRACTS, ILLUSTRATIONS, QUERIES, SUGGESTIONS, &c.

RELATING TO THE

*History or the Art of Medicine, and the Auxiliary Sciences.*

---

### *On Vomiting: being an Account of a Memoir of M. Magendie on Vomiting, read to the Imperial Institute of France on the* 1*st of March*, 1813.

THIS memoir treats of a physiological truth which for a century and a half past has been alternately adopted and rejected, acknowledged and denied, established and forgotten, and which M. Magendie has at last founded on proofs so irrefragable that it is completely established, and must henceforth be considered as a point of doctrine beyond the reach of every objection.

How is vomiting performed, and what are the means employed by nature for that act, so apt to disturb the health, and in many cases so well adapted to re-establish it? Such is the question which occupied the indefatigable and ingenious author of the memoir of which we have to give an account. He has not considered it with reference to medical practice, convinced that in what way soever it is produced, its necessity, indications, and effects, must continue the same in cases of disease. He has treated it as a skilful physiologist and judicious experimenter; and if we cannot ascribe to him alone the idea and the entire solution, it is just to say that without him it would still have remained a problem undecided.

Nobody doubted till towards the middle of the 17th century that vomiting was produced by the simultaneous contraction of the muscular fibres of the stomach, supposed by anatomists to exist in that organ upon no very strong evidence. M. Magendie says, in his memoir, that Chirac appears to have been the first who entertained the contrary opinion, and who advanced that the diaphragm and abdominal muscles are the essential agents; but we have found that Bayle entertained the same opinion long before that physician, and that he confirmed it by experiments, which, if they they were really made, must deprive Chirac of the priority, without injuring the proofs by which he confirmed his opinion. Senac informs us that Bayle, having caused a dog to swallow an emetic, made a deep incision opposite to the stomach, through which he introduced his finger while the animal was in the act of vomiting, and found by repeated trials that the stomach was not in motion. He found that the whole action was produced by the diaphragm and abdominal muscles, the most powerful of which, according to Senac, are the two transverse muscles, the only ones which have a semicircular direction, and which are capable of forming those hollows that appear in the belly in the act of vomiting. It is needless at present to discuss this subject.

The system of Bayle, or of Chirac, had its partisans; but it met likewise with opponents. These indeed could not but be numerous at a time when it was believed that the food was triturated in the human stomach in the same way as it is in the gizzards of birds.

On this occasion there occurred a pretty keen discussion between two members of the Academy of Sciences, Litre and Duverney; one of whom employed inaccurate reasoning, the other inconclusive experiments; and neither was able either to convince the followers of Chirac, or persuade his antagonists. Lieutaud and Haller, almost at the same time, put themselves at the head of the last party. They endeavored to prove that vomiting is exclusively performed by the stomach, and that it is independent of the diaphragm and abdominal muscles, which in their opinion only concur accidentally with the action of the stomach. Lieutaud observed that the action of the diaphragm and abdominal muscles being subject to the will, vomiting ought to be voluntary if it was occasioned by any such action; yet this is the case only in a small number of instances. Haller opposed the opinion of Chirac in order to strengthen his own system of *irritability*, under which he wished to arrange all the phenomena of animal organisation.

Wepfer took the same side, and he deceived himself still more than his predecessors; for he had recourse to experiments, and was misled by the results. He employed poisons by way of emetics, which excited in the stomach, sometimes in its place, sometimes out of the body, movements which he considered as muscular actions, though they were only the effect of that contraction which takes place in living substances when attacked by corrosives.

The high reputation of Haller, and the influence of his works, almost effaced the very recollection of the true notions of of vomiting which had been occasionally perceived; and during fifty years it had been uniformly taught and believed that vomiting is produced by the stomach alone, till M. Magendie turned his attention to the subject, and resolved to subject it to experiments so rigid, and so often repeated, as to put the question beyond dispute, and render the conclusions classical, both in books and the schools.

We think proper to mention here that M. Richerand, an esteemed professor and author, convinced by facts exhibited before him, as well as before us, has introduced them into his treatise of Physiology, and has employed them to explain the notion of vomiting which he has embraced.

It is principally by the faithful account of these facts that M. Magendie so greatly interested the Class, already accustomed to esteem his talents and appreciate his discoveries. It is by recalling them to our colleagues that we hope to interest them in our turn.

We have not to notice simple conjectures, or slight and trifling attempts, from which systems have been too often built, and opinions formed respecting the most difficult points. Never, perhaps, were experiments more multiplied on the same object, or more scrupulously conducted, or with more exactness. They have been repeatedly made before us. We carried to them a considerable portion of doubt, perhaps even of incredulity, without, however, suspecting the well-known veracity of their author. We have seen, examined, handled, and we declare that our conviction is full and complete.

All the experiments which we witnessed were made upon dogs, because they are the animals most subject to vomiting. Tartar emetic was almost always employed to produce vomiting, not by way of injection or deglutition, but by introducing it into the jugular vein, as is done by the veterinary schools of Denmark. And it is worthy of remark, that tartar emetic, when swallowed by the animal, often does not occasion vomiting in half an hour; but when introduced directly into the circulation, it produces vomiting in one or two minutes. We have reason to be astonished at this constant and irresistible tendency of tartar emetic to produce vomiting, so that wheresoever it is applied it always produces this effect.

As Bayle, Chirac, and Duverney had announced, M. Magendie made us perceive by the touch that during the act of vomiting the stomach remains in a state of inactivity, and that it is the diaphragm and abdominal muscles which produce the evacuation of that organ. During this first experiment, repeated several times upon large dogs in the abdomen, abdomen, of which an incision had been made large enough to admit two fingers, we perceived that at each strain of the animal our fingers were pressed upon from above by the liver pushed down by the diaphragm, and from below by the intestines which the abdominal muscles pressed, while the stomach, emptying itself without any sensible motion, did not appear to diminish in volume. This last singularity, already observed and announced to the Class by M. Magendie, is occasioned by the presence of air, which takes the place of the food as it is thrown out of the stomach, and which, being introduced through the œsophagus during the long inspirations which precede vomiting, keeps the stomach always sufficiently distended not to escape the compressing action of the surrounding parts.

We know that it is easy to swallow air. Some people amuse themselves by swallowing it, and thus swell out their stomach till it resounds like a drum when struck. There can be no doubt that a great deal of air is swallowed during vomiting, without which vomiting would be exceedingly painful, as happens in cases of poison by corrosive substances, when the stomach is contracted, and no longer capable of admitting that fluid. M. Magendie intends soon to read a memoir on this subject to the Class, and we ought not to anticipate what has become his legitimate property. We shall observe the same silence with respect to the conspicuous part which the œsophagus takes in the act of vomiting, because M. Magendie is drawing up a memoir on the subject.

In a second experiment, made upon the same dogs which had served for the preceding, the incision of the belly being increased, and the stomach drawn out of the body, it was still easier for us to be convinced of its want of motion, and to perceive the inaccuracy of what Haller had advanced respecting its peristaltic movement. In this state the stomach, filled with air which had been drawn in some moments before the act of vomiting, was distended like a balloon; but no farther vomiting took place, nothing but ineffectual nauseas, because the stomach being out of its place could no longer be acted upon by the surrounding organs.

M. Magendie announced in his memoir that by pressing upon the stomach thus removed out of the body with the two hands, so as to imitate in some measure the action of the diaphragm and abdominal muscles, vomiting was always produced. And this constitutes one of the most conclusive arguments in favor of the opinion which he embraced; but though the dog subjected to this experiment vomited without having taken any emetic, and exhibited the nausea, and other other symptoms which characterise vomiting, the column of air did not enter and take the place of the ejected food. This shows us that other conditions besides the mere pressure of the stomach are necessary to produce vomiting. This experiment revealed to M. Magendie the principal of these conditions. When he held the stomach in his hands without compressing it, he perceived that when he drew it too far out of the belly he immediately produced nausea and vomiting. He conceived that it was the stretching of the œsophagus which produced this double effect; and he took advantage of this discovery to make dogs vomit at pleasure which had taken no emetic, or to hasten vomiting when the emetic did not act with sufficient promptness. It was only necessary in either case to agitate the stomach, and draw the œsophagus a little, to produce immediate vomiting. It is easy to perceive here the effect of those profound inspirations which, as well as nausea, precede vomiting, and by means of which, the diaphragm embracing the œsophagus between its pillars, draws it along with it towards the intestines, and makes it undergo those tractions which M. Magendie has so happily imitated. This explains why in the palsy of the œsophagus there is no vomiting, and why it is so difficult to produce it after cutting the pneumogastric nerves.

If we examine a person just going to vomit, if he does not succeed after a strong inspiration, we see him repeat it again and again, and multiply the movements of expiration, which are always more irregular. By this means the diaphragm agitated up and down gives to the œsophagus that agitation without which, in all probability, vomiting would not be produced.

It is well known that vomiting often takes place without all those efforts. This is an objection which may be started against either opinion. But, besides that we do not speak of those individuals, who, from the frequent practice of vomiting, have acquired the habit of it, we must distinguish, in infants at the breast, for example, the regurgitation of vomiting; and, in persons who ruminate, the voluntary and tranquil act of bringing from the stomach to the mouth the food to be swallowed a second time, from the painful and involuntary act of vomiting. Besides, in persons who ruminate, as has lately been observed by one of your commissioners in a young man of 24 years of age, the return of the food to the mouth is preceded by a kind of noise, sometimes pretty loud, which announces the instantaneous agitation of the œsophagus, produced by the diaphragm, and the no less prompt action of the œsophagus upon the stomach.

This agitation of the œsophagus is not confined to the alimentary canal, properly speaking; the branches of the par vagum, and of the great intercostals which cross around it, must participate in this movement.

We observed above, that as long as the stomach of dogs laboring under an emetic was out of the body, no vomiting took place, but only nausea; but when the stomach was restored to its place, vomiting immediately followed. The next point to be determined was, if the action of the abdominal muscles be absolutely necessary to produce vomiting, as was the opinion of Chirac and his adherents. These muscles were removed from a robust dog, and an emetic being injected, he vomited apparently with as much facility as if that operation had not been performed, which reduced the covering of the abdomen to the peritoneum, and to a few transverse muscular fibres which it was impossible to remove. M. Magendie made us remark in this case the great tension of the linea alba, during the nausea and vomiting; and we conceive that this species of cord stretched along the abdomen may be sufficient to keep the intestines in their places, and to prevent them from escaping from the energetic action of the diaphragm, which in some of the experiments even tore the peritoneum in several places.

One of us had made an analogous observation, but without drawing the same consequence, upon a soldier, the muscles of whose abdomen had been removed or destroyed by the action of a large cannon-ball; so that after his cure the stomach in all its positions might be seen through the transparent peritoneum. This soldier, during his cure, was frequently troubled with vomiting, to which the abdominal muscles could not contribute, as they were wanting altogether; yet he vomited with as little difficulty as before his wound.

The experiment above related, which was first thought of by M. Magendie, proves that it is the diaphragm which acts with the greatest efficacy in vomiting, and that the abdominal muscles serve scarcely any other purpose than to confine the viscera floating in the abdomen, and to oblige them to re-act in a contrary direction. But when the action of the diaphragm is carried too far, and when the inspirations are too profound and too long, then instead of vomiting we have alvine evacuations, doubtless because the œsophagus is too much pressed upon by the diaphragm to give free passage to the substances which endeavor to escape from the stomach.

When, on the contrary, the diaphragm can only act feebly, and solely for the maintenance of respiration, as happens when the phrenic nerves are cut, then how strong soever an emetic is administered, nothing more takes place than successive nauseas, and very seldom vomiting, notwithstanding the most violent contractions of the abdominal muscles.

One of the commissioners having invited M. Magendie to cut the phrenic nerves on both sides of a dog still vigorous, whose abdominal muscles had been removed, and to make him swallow a gros (72 grains) of red oxide of mercury, the animal was very much agitated, and had nauseas, retchings, and painful alvine evacuations, but did not vomit. M. Magendie intends speedily to state the observations which he made on this occasion.

Most of these experiments prove sufficiently that the stomach is entirely passive in the act of vomiting, and that the principal effect is produced by the diaphragm. Those that follow go still farther, since they demonstrate that vomiting may take place without the stomach. They were repeated three times in our presence with the same result.

M. Magendie having cautiously (in order to avoid hemorrhages) made a ligature on each of the orifices of the stomach, removed that viscus altogether, and, after having sewed up the wound in the belly, administered an emetic. In less than two minutes the dog exhibited all the symptoms which precede vomiting. We may even say that he actually vomited, for he threw out with effort and violent nausea the mucus of the œsophagus. Thus it appears that vomiting may in some measure take place without the stomach. It appears, then, that as far as vomiting is concerned the stomach is nothing but an inert bag, containing matters destined to be thrown out. And what other part in vomiting is it possible to ascribe to those schirrous stomachs whose coats have acquired some inches of thickness, and a hardness approaching to that of cartilage?

We have only another experiment to notice, and it is the most extraordinary and the most decisive of all those which we have seen.

In the place of the stomach, which had been cut out of several dogs, M. Magendie substituted a small hog's bladder, almost of equal capacity, to the neck of which a canula of caoutchouc had been adapted, which was thrust into the œsophagus below the diaphragm, and kept in its place by a thread. These dogs were made to swallow water tinged yellow, with which the bladder was filled according as deglutition took place. The opening of the belly having been sewed up, an emetic was introduced into the jugulars. Nausea took place in a short time, and the animals vomited the yellow water precisely as if it had come from a real and living stomach. The wound in the belly being laid open, we we easily observed at each strain the air descending in a current into the bladder, and distending it as if it had been a real stomach, which is not the least curious circumstance attending this experiment.

It only remains for us to submit to the Class some reflections which M. Magendie did not think it necessary to add to his memoir, though he did not fail to make them as well as ourselves, on the question whose destiny he has thus finally fixed.

These experiments prove not only that the stomach is passive in vomiting, they lead us to a more important result, which throws new light upon the nervous energy, that wonderful energy which constitutes the whole of our being, the mysteries of which it is so much our interest to penetrate. We may deduce from the result of these experiments that the principle, the prime mover of all those movements which produce vomiting, has its source in the seat of the nervous energy itself; for we cannot otherwise explain how an emetic, which produces no action on the stomach, determines the contraction of the diaphragm and abdominal muscles. We cannot have recourse here to those sympathies which have been so much abused in physiology, by advancing that the contraction of the stomach draws along with it by sympathy that of the muscles just mentioned. It is obvious that an emetic can only produce its effect by re-acting from the stomach upon that place of the seat of the nervous energy, where the principle of the contraction of the diaphragm and abdominal muscles resides. It is the affection of that part which is the immediate cause of vomiting. If the nerves, by which the diaphragm and the abdominal muscles receive the impression of it, were cut, the patient would have the same desire to vomit, and would have the sensation of vomiting without vomiting in reality. This is proved by the suspension of vomiting in M. Magendie's experiments on cutting the phrenic nerves. On the other hand, though these nerves, and all the rest of the body, remained untouched, if that portion of the seat of the nervous energy were disorganised, no emetic could give the animal either a desire to vomit, or produce in him the sensation of vomiting.

We have here a particular and very remarkable application of that general truth demonstrated by M. le Gallois, namely, that the seat of the nervous energy (the brain and spinal marrow) is the sole source of all the motions which take place in a living animal, and that no part can move without a particular and anterior modification of that part of the nervous energy by which it is animated. The obstinate vomiting which in many cases accompanies apoplexy, and

 which which had been ascribed to indigestion, had been already pointed out by M. le Gallois as a phenomenon entirely unconnected with every affection of the stomach, and totally depending upon that of the brain.

It remains to be known how an emetic introduced into the stomach can affect the seat of the nervous energy in a manner so as specifically to produce vomiting. Is it by irritating the nerves of the stomach? or is it absorbed, introduced into the blood, and transported by the circulation? Perhaps both of these modes of transmission take place, according to circumstances. The vomiting which we observe sometimes after cutting the nerves of the eighth pair, and which appears to be occasioned by the irritation which the superior segment of these nerves experiences, seems to favor the first mode of action; while the experiments of M. Magendie producing vomiting even in animals deprived of their stomach, by injecting an emetic into the blood-vessels, seems equally favorable to the second mode. His preceding experiments on the effect of upas, experiments made in concert with M. Delille, strengthen this last opinion. They prove that upas occasions those dreadful convulsions which so speedily destroy life, only when it is absorbed into the mass of the blood, and transported directly to the spinal marrow. It is very probable that almost all substances that have some effect on the animal economy act in this manner. This opinion leads us to views entirely new respecting the mode of action of most medicines and poisons.

Another question remaining to be answered, is to know the precise part of the brain or spinal marrow on which the efforts of vomiting depend. M. le Gallois has proved that the principle of the movement of inspiration is seated in that portion of the medulla oblongata which gives origin to the eighth pair of nerves. If we consider that the efforts of vomiting are executed by the muscles of respiration, that the nerves of the eighth pair supply the stomach as well as lungs, and that the disorder of the medulla oblongata in apoplexy occasions vomiting, it will be rendered pretty probable that the efforts of vomiting are situated not far from those of respiration, if they have not the very same position. But it would be of importance to determine the point by direct experiments. Now that the general seat of the nervous energy is well determined, and clearly defined, one of the greatest objects of physiology is to know precisely the function peculiar to the different portions of that seat. Such objects deserve the attention of such accurate experimenters as MM. le Gallois and Magendie; and those experiments, which they have already made so successfully, induce us to hope that they will will advance still farther in a career in which they know by experience that they are likely to meet with honor, glory, and reputation.

To conclude, we think, 1. That M. Magendie, to whom the Class has already given with so much pleasure proofs of its esteem and satisfaction for the experiments previously communicated, deserves new ones for those which he has just presented.

2. That his memoir on vomiting, destined to be ever after cited in physiological works, is worthy in the first place of being mentioned in the history of the labors of the Class, and of an honorable place in its memoirs.

3. That M. Magendie ought to be invited by the President to give to his experiments the farther developements of which they are susceptible; and to demand, if he thinks proper, a reimbursement of the expenses which he may have incurred, or may still incur, in the further prosecution of the subject; for we expect that he will examine with particular attention the phenomena of vomiting in birds, and other animals destitute of a diaphragm.

(Signed) CUVIER,

PINEL,

HUMBOLDT,

PERCY, *Reporter.*

The Class approves of this Report, and adopts its conclusions.

Certified conformable to the original.

The Perpetual Secretary, Knight of the Empire,
G. CUVIER.

---

*Vaccination in India.*

The English have introduced the blessings of vaccination among all descriptions of people in Hindoostan; by which means the lives of thousands and tens of thousands are annually preserved. In this humane undertaking the Brahmins have risen superior to prejudice; and under their extensive and powerful influence, all other casts of Hindoos have adopted the practice. Many letters on this subject from eminent Brahmins to medical gentlemen in India do them honor; they contain the most liberal sentiments, and have been followed by a corresponding practice. *Mooperal Streenivaschary*, a Brahmin, thus writes to Dr. Anderson, at Madras, on vaccine inoculation:—

"I beg leave to observe, for the information of the natives of this country, that I have perused the papers which you have published on that wonderful, healthful, and immortal vaccine matter, discovered on the nipples and udders of some some cows in England, by that illustrious physician, Dr. Jenner; whereby the loathsome, painful, and fatal small-pox has been prevented from seizing the many of our fellow-creatures in India, as well as in Europe.

"I am an eye-witness, as well as many others, that numbers of children here have been inoculated with vaccine matter, without any injury or blemish whatsoever, excepting a small spot at the place where the matter is applied, which is commonly in the arm. It is therefore greatly to be wished that an intimate knowledge of this wonderful discovery may be acquired by the natives of this country, so as to enable them to preserve the lives of the rich and honorable, as well as those of low casts. On this account, it might be useful to remove a prejudice in the minds of the people, arising from the term cow-pock, being literally translated *comary*, in the advertisement which has been published in our Tamul tongue; whereas there can be no doubt that it is a drop of nectar from the exuberant udders of the cows in England, and no way similar to the humor discharged from the tongue and feet of diseased cattle in this country.

(Signed) MOOPERAL STREENIVASACHARY."

As vaccination is now so generally adopted in Hindoostan, and likely to become a universal blessing in that populous part of the globe, it may be satisfactory to mention the following singular fact, respecting the antiquity of vaccination in India, taken from the Asiatic Register for 1804; which is altogether a curious and authentic addition to a subject so interesting to humanity.

"The fact stated in the following translation of a written memorandum from the Nabob Mirza Mehady Ali Khan, who was long resident at Benares, that the effects of vaccination have been known for a great length of time in that celebrated quarter of India, is referred to the investigation of those who have the opportunity and ability, since they cannot want the inclination, to prosecute so interesting an inquiry. The undoubted intimation of this fact, that vaccination has been practised among the worshippers of Bowannee, will not detract an iota from the merits of the Jennerian discovery; the fortuitous and happy circumstance that led to the discovery in Europe, has been unquestionably and most satisfactorily proved, whilst the anxiety, study, perseverance, and indefatigable exertions, which have been applied by its benevolent professor to ensure the conviction of the world, in the unbounded benefits of the discovery, have entitled him to the lasting gratitude of mankind. The full ascertainment of the fact will only go to afford an additional instance of primeval oriental knowledge; whether acquired or accidental, is to be hereafter proved: it will only open an additional neglected mine for the curious and the learned; and will be another proof that the East has been the seat of wisdom, 'where learning flourished, and the arts were prized;' however much the neglect with which this knowledge has been treated in this country, may reflect upon the modern degeneracy, or the prejudices of the Indian character; which may, however, be all accounted for, from the effects of the various revolutions, to which their country has, for so many ages, been a prey; leaving thence room to the liberal construction of the unbiassed of every nation to conclude, that before the introduction of a foreign sway into Hindoostan and the Deccan, its Hindoo inhabitants were versed in the arts and sciences, far beyond the other parts of the world, at the same remote period of time.

"Translation of a written memorandum from the Nabob Mirza Mehady Ali Khan.

"During the period of my abode in the district of Benares, my eldest son being taken ill of a bad kind of the small-pox, and my friends interesting themselves for my comfort and his relief, one of them, named Slookum Chund, a Hindoo, pointed out to me that there was in the city of Benares, one Alep Choby, a Brahmin, from Oude, whose practice was chiefly confined to this malady. Him, therefore, I lost no time in sending for to the town of Ghazeepoor, where I dwelt; and he arrived on the ninth day of the eruption; on seeing which, he observed that if the eruption had not taken place, he would have endeavored to facilitate and render it easy; but that now it was too late. On asking Choby what his process was, he said, 'From the matter of the pustule on the cow, I keep a thread drenched, which enables me, at pleasure, to cause an easy eruption on any child; adoring, at the same time, Bowannee, (who is otherwise called Debee, Mata, and Seebla, and who has the direction of this malady,) as well in my own person, as by causing the father of the child to perform the like ceremonies; after which I run the drenched string into a needle, and drawing it through between the skin and the flesh of the child's upper arm, leave it there, performing the same operation in both arms; which always ensures an easy eruption; on the first appearance of which the child's father, or guardian, renews his worship to Bowannee; and as the animal this goddess rides on is an ass, it is customary for such parent or guardian to fill his lap with grain, which an ass is sent to eat up; these observances ensure the propitious direction of Bowannee, so that only a very few pustules make their appearance; nor does any one die under this process.'—Thus far did I learn from Alep Choby.

"Upon referring on this subject to a native well versed in the learning and customs of the Hindoos, he told me that the practice thus described by Choby was not general among them, but confined to those who were attached to the worship of Bowannee, and adored her with implicit faith: and upon my asking the person whether he was aware how the matter of the pustule got from the cow, and whether all cows had such pustules, or only those of a certain description; he answered, that on these points he possessed no information; but had certainly understood that the cows had these pustules break out on them, and that from the matter thereof children were infected; acknowledging, however, that he spoke not this much from ocular knowledge, but from report."

---

Mr. John Underwood, senior, who resided many years at Madras in a medical capacity, frequently corresponded with Surfojee, *Rajah of Tanjore*, a most amiable benevolent character, and particularly fond of studying anatomy. Mr. Underwood sent the rajah a body, where the heart and every artery and vein were carefully injected with colored wax: this preparation would give him a correct idea of the course of circulation, and the insertion of several muscles. The rajah was much gratified by a present which enabled him to pursue his studies with increased delight, and rendered him more useful in his sovereignty. The following letter, in acknowledgment of this valuable present, was written by the rajah to Mr. Underwood, which, in a Hindoo prince, indicates a mind unusually liberal and enlightened, sufficient to encourage a lively hope towards the advancement of literature, art, and science, extending ultimately, perhaps, to establish Christianity in that part of Hindoostan where there are already several protestant churches. For Surfojee, Rajah of Tanjore, was the friend and patron of Swartz, for near half a century the apostolical missionary of Coromandel, whose prudent zeal truly blended the wisdom of the serpent with the innocence of the dove. The Hindoo sovereign shed tears at the death of his venerable Christian friend, and covered his remains with a splendid pall of gold brocade. On the establishment of the Native Hospital at Madras, in 1799, under the immediate care of Mr. Underwood, senior, this benevolent sovereign sent two thousand pagodas, about eight hundred pounds sterling, to assist the institution.

*Letter from Surfojee, Rajah of Tanjore, to John Underwood, Esq. at Fort St. George.*

I received your letter some time ago: the contents of it have yielded me inexpressible pleasure. The box and the book book alluded to in the letter, have likewise been safely received.

The human body, of which the origin appears to have been wrought by the Supreme Being himself, the frame of which is supposed to have afforded satisfaction even to its maker, has been the chief object of my long imitation and inquiry. The books with which I have been conversant, have spread before me but a faint light on this topic: hence I need not say that the preparation which you have sent to me, has afforded me the greatest pleasure; especially as I have long been desirous to see one of this kind, the receiving it so unexpectedly from you, has redoubled my satisfaction. Upon examining every part of it, I found the muscles to be well preserved; and it is worthy of the inspection of every lover of philosophy.

The book of anatomy which you have been so obliging as to send me, is also well calculated for the students to profit by. I request you will accept of my thousand thanks for the trouble you have taken in forwarding me the above things, which are very useful and pleasing to

*Tanjore*, *July* 5, 1806. SURFOJEE, *Rajah*.

Not being myself sufficiently competent to elucidate the subject of medicine, as practised by the natives of India, I requested Mr. Underwood to give me some account of the general mode of treatment in that part of the world; in consequence of which he favored me with the following statement, which I introduce with great satisfaction, from its filling up a desideratum I could not have supplied from my own knowledge.

"It appears to Europeans that the natives of India are extremely ignorant in the practice of physic: they have many remedies, chiefly roots and herbs, which are generally given in the form of powders. The practitioners are poor men of a particular cast, who sit by the side of the high roads and market paths, with small boxes, containing various kinds of powder, which is administered with particular instructions, and a promise of cure in a specific number of days. In all complaints they enforce abstinence, seldom allowing the patient any other nourishment than their conjee, or rice gruel. In certain diseases they give cinnabar, occasionally with success; but the improper use of it frequently causes ulcerations to spread to a very great extent.

"The natives are extremely bigoted to their own remedies, which, without improvements or alteration, are handed down from father to son through succeeding generations. They, therefore, seldom apply for the assistance of Europeans until the the case appears hopeless from their own prescriptions. They do not bleed, nor perform any surgical operation, unless the removal of a part partially divided. All cases of fractures and dislocations are consigned to the potters, a cast of people abounding in Hindoostan, for making the water-jars and cooking utensils, of red clay, so universally used. The potter places the limb of his patient in what he considers the best situation, and then covers the part affected with moist clay: this, when dry, fixes the limb; and under such treatment simple and compound fractures often do well, but, as may be expected from this process, distortions and stiff joints are more frequently the consequence.

"For spasmodic affections, the natives of India generally apply the juice of the milk-bush (*Euphorbia*, Linn.) to the parts affected, which acts like a blister: in more serious cases they use the actual cautery. From this cause it is common to see horses, oxen, laboring men, especially palankeen bearers and porters of heavy burthens, marked in many places by a hot iron. Notwithstanding the liberal mind and singular propensity of the Tanjore sovereign, already mentioned, it cannot be expected that these medical practitioners should in general acquire any accurate knowledge of anatomy, and the heat of the climate operates powerfully against their possessing any extensive information from dissection: much, however, may be acquired from preparations.

"Although I have no high opinion of the general mode of practice among the natives, yet in a few instances I should give a preference to their remedies, particularly in the ophthalmia or sore-eye of India: the inflammation frequently runs so high that the sight is destroyed, unless by some active means, the affection, so deeply rooted, can be removed. This, I think, is best done by an early application of what is called at Madras the '*country remedy*,' which is a thin paste, made by burning a little alum on a hot iron, and mixing it with lime-juice, by a spatula, into a paste. This is applied over both eye-lids, to the extent of the circle of the orbit, at going to rest, and washed off in the morning with a decoction of tamarind leaves. This I consider the best and most certain remedy for a disease that so repeatedly causes blindness. A surprising number of the natives are entirely blind, especially among the poor.

"I have often seen a Mahomedan practitioner perform the operation of removing a cataract. He made a small puncture with the point of a lancet, immediately behind the iris, into which he introduced a particular instrument, so guided as to depress the cataract. This operation I prefer to to any other mode yet practised, as it occasions less injury to the eye."

The preceding appears to be a clear and brief statement of medical and surgical practice among the natives of Coromandel, and I believe the same system is generally, or nearly, adopted throughout Hindoostan. When with the Mahratta army during Ragobah's campaign in Guzerat, I had frequent opportunities of knowing the high estimation in which the English physicians were held both by Hindoos and Mahomedans, when they thought themselves seriously ill, or wished to procure their advice even for their females, whom, indeed, they were not often permitted to see, but formed their judgment of the disorder by feeling the patient's pulse with the arm admitted through a perforated curtain. It certainly would be no easy matter to persuade a Brahmin to mingle Peruvian bark, or any other medicine, with wine or distilled spirits; but to take the drug in simple water, or compounded with any ingredients he was accustomed to, would not be attended with difficulty. As to the other casts in general, provided they are persuaded the prescription is to effect a cure, or prove a stimulus, they wave the ceremony of being very particular in their inquiries.—*Forbes's Oriental Memoirs.*

---

### *Goderneau's Powder.*

This powder is a nostrum used at Paris, and reprobated by the regular physicians as too drastic for the human frame. Dr. Marcet examined and found it to be a preparation of mercury. Each parcel given as a dose contains xij grains French. The color is not so white as that of calomel. Examined with a lens, small globules of metallic mercury are discernible, and also some reddish particles, which are red precipitate. It is wholly volatile at a low heat like calomel, but volatilization separates it in a manner which proves it not to be homogeneous, for the vial in which it is sublimed is marked with three distinct zones, white, red, and grey. Water does not dissolve any sensible portion of this powder. Nitric acid dissolves the whole of it, and nitrate of silver poured into the solution, lets fall a quantity of muriate of silver corresponding to about nine grains of calomel. The remaining three seem composed of about one and a half metallic mercury, and one and a half red precipitate. By triturating the above substances in the above quantities, a compound very like Goderneau's Powder was produced, but rather more uniform in its appearance. This is a very rude preparation of mercury, and its use should not be encouraged. Its effect is to produce a very disagreeable sensation in the stomach, stomach, and afterward to increase the appetite. It also purges, and sometimes vomits. The Chevalier Goderneau was a military man, and a knight of St. Louis, but no chemist; and since the revolution has swept him from the earth, the care of preparing this powder has devolved upon his sister, an old maiden lady, who from time to time swallows large doses of it for a sore foot, which has the advantage of always being about to heal.

---

### *Establishment for Warm Baths and Douches at Paris.*

The establishment of artificial mineral waters at Paris, is very extensive and useful. All the known mineral springs in Europe are there prepared according to the best analysis of modern chemistry; and in sufficient quantity for drinking, bathing, or douches. The climate of Paris is colder in winter, and warmer in summer, but it is much less damp than that of London: and during five months of the year a continuance of fine weather is much more to be depended upon. Almost every evening, from the beginning of May to the middle of September, all the public walks which abound in and near that city are crowded with persons, who, very lightly clad, remain walking or sitting in the open air, till eleven or twelve o'clock at night; a system of life which no inhabitant of England could follow eight days consecutively without suffering by it. Warm bathing is therefore (why?) not of such general use or benefit in England as in France. There are few places where nature has done so much in favor of warm bathers as at Bath; and art has done almost every thing except taking advantage of those very waters which have caused the prosperity of all the rest. The baths, however, have been considerably improved of late years, but still there does not exist at Bath so efficient a Douche as those of the artificial establishment at Paris. The strongest douche at Bath falls from a perpendicular height of fourteen feet. The highest douche at Paris falls from 32 French feet, a height which is wholly arbitrary as to medical effect, and is merely founded on this physical fact, that the weight of a column of water 32 feet high, is equal to the pressure of the entire atmosphere upon a basis equal to that of this column of water. But as the force of falling water increases in the ratio of the square of the perpendicular height of the pipe through which it falls, the douche at Bath has not one-fourth part of the force of that in Paris, and the difference of their effect is very great. A small expence might rectify this deficiency at Bath, and establish douches of any height; and which in their application might be proportioned to the sensibility of the patient.

